# Differences in sensitization rates between children with Chronic Eczema and general population: Can they provide insight into significance of particular haptens for the disease?

**DOI:** 10.1186/2045-7022-5-S1-P5

**Published:** 2015-03-11

**Authors:** Radoslaw Spiewak, Danuta Plichta

**Affiliations:** 1 Jagiellonian University Medical College, Dept of Experimental Dermatology and Cosmetology, Krakow, Poland

## Background

Contact allergy is present in 58-67% children with chronic eczema, yet some children with contact allergy never develop eczema. To assess the significance of contact allergy in eczema, rates of sensitization to each hapten should be compared between eczema patients and general ("healthy") population. It seems reasonable that haptens with higher odds ratios and attributed risk for sensitization (i.e. better discriminating between ill and healthy) are "more important" in eczema, whether cause or result. The present study was aimed at estimating these indices for selected haptens.

## Method

A systematic review of published data on the prevalence of sensitization to various haptens in eczema patients and the general population of children. The data were extracted and pooled to calculate estimated odds ratios (eOD) and estimated attributable risks (eAR).

## Results

Preliminary analysis has shown that these indices differ substantially for various haptens. The highest eOD was found for formaldehyde: its value of 31.3 means that contact sensitization to this hapten was 31.3 times more frequent in children with eczema than in healthy ones. As 9.4% children with eczema and only 0.3% healthy children were sensitized to formaldehyde, the computed eAR for this hapten amounted to 9.1%. Other analysed haptens were Myroxylon pereirae (Balsam of Peru, eOD=21.6; eAR=10.3%), wool alcohols (lanolin, eOD=17.3; eAR=11.4%), MI/MCI (Kathon CG,

eOD=15.0; eAR=19.6%), chromium (eOD=8.3; eAR=10.9%), fragrance mix (eOD=6.9; eAR=10.1%), nickel (eOD=2.3; eAR=10.9%), PTBF resin (eOD=1.4; eAR=0.4%) and neomycin (eOD=0.9; eAR=-0.3), to name just a few examples.

## Conclusion

Sensitization rates to certain haptens are considerably higher among children with eczema than in healthy children, while there is no difference for others. The proposed approach offers a valuable insight into significance of particular haptens and may help at selecting haptens for routine patch testing in children with eczema.

